# Capillary hyperpermeability syndrome: A fatal complication of acute leukaemia: Case report and review

**DOI:** 10.1016/j.amsu.2022.103336

**Published:** 2022-02-04

**Authors:** Hajar Benzekri, Amine Bouchlarhem, Nour el houda lamassab, Meryem Jabri, Fadoua Jabrouni, Chaimae Daoudi, Nouredine Oulali

**Affiliations:** a Faculty of Medicine and Pharmacy, Mohammed Ist University, Oujda, Morocco; b Department of Emergency, Mohammed VI University Hospital, Mohammed I University, Oujda, Morocco

**Keywords:** Acute leukemia, Capillary leakage, Clarkson syndrome- generalized oedematous syndrome

## Abstract

**Introduction:**

The capillary hyperpermeability syndrome is a rare disease that should be suspected in the presence of recurrent generalized edema without obvious cause, which may be idiopathic or secondary.

**Case presentation:**

In this case, we report a Clarkson syndrome secondary to an acute leukemia affecting a 4-year-old child admitted to the emergency room in respiratory and hemodynamic distress with a generalized oedematous syndrome and a bone marrow failure syndrome. Laboratory tests concluded that the patient was suffering from an acute lymphoblastic leukemia, hypoalbuminemia, pericardial effusion, and the absence of any other cause that is in favor of a capillary leak syndrome.In spite of the filling and the introduction of drugs, the cardio respiratory arrest could not be recovered and the child died 24h after his admission.

**Discussion:**

It is a rare pathology described for the first time in 1960, generally secondary to a pathological state and more rarely idiopathic, to be evoked in front of clinical and biological parameters which are hypoalbuminemia, hemiconcentration and hypoperfusion, after having eliminated a sepsis in the first place.The treatment is based on the management of the acute phase by filling with crystalloids, drugs or even steroids, and as a preventive treatment of relapses immunoglobulins or theophylline are used.

**Conclusion:**

The evolution can be quickly fatal, that's why it is necessary to know how to evoke this syndrome in front of a similar clinical presentation.

## Highlights

• Capillary hyperpermeability syndrome is a rare cause of generalized edema.
• The diagnosis is made on the basis of hypoalbuminemia, hemoconcentration, and hypoperfusion.
• A monoclonal gammopathy type igG is rarely found in children unlike in adults.
• Relapse prevention remains a very important part of treatment with immunoglobulin, theophylline or anti TNF alpha.

## 1 Introduction

Capillary hyperpermeability syndrome (or Clarkson Syndrome) is a rare syndrome that can be idiopathic or secondary, as in the majority of cases, its diagnosis is retained after eliminating the various differential diagnoses essentially sepsis, if we have a hypo albuminemia, hemoconcentration and a peripheral hypoperfusion.

The management of the acute phase is based essentially on the management of the shock with a strict monitoring of the hemodynamic constants, and to avoid relapses, the administration of immunoglobulin, theophylline/terbutaline or even anti TNF alpha is recommended.

## 2 Case presentation

We report the case of a child aged 4 years and 8 months with no medical or surgical history, the patient only reported 3 spontaneously resolved episodes of edema during the last 3 months, he was admitted for the management of profound asthenia associated with generalized edema.

The clinical examination on admission found a shocked patient with a blood pressure 75/39 mmhg a Heart rate at 140bpm, unstable on the respiratory plan with a spo2 at 64% on room air, presence of an edematous syndrome made of: edema of the bilateral lower limbs reaching the thighs, ascites, cervical-facial edema with a weight of 23 kg, the urine bandellette is negative.

The physical examination found a generalized cutaneous-mucosal pallor associated with a hemorrhagic syndrome made of oral hemorrhagic bullae and ecchymosis at the dorsal, scrotal and plantar level, the abdominal examination found a hepatosplenomegaly, the rest of the examination found cervical, axillary and inguinal adenopathies bilaterally.

The blood count performed on admission showed: hemoglobin 3.9 g/dl (normal value 12–16 g/dl); platelets 40 G/l (normal value 150–450 G/l), white blood cells 387 220/mm3 (normal value 3000–10000/mm3). The myelogram showed 87% blasts, and immunophenotyping showed B lymphoblastic acute leukemia.

The biochemical workup found: ASAT 184 IU/l (Normal value 0–34IU/l), ALAT 16IU/l (Normal value 0–54IU/l), LDH 322 IU/L (Normal value 35–265IU/L), collapsed albumin at 26g/l (Normal value 40–70g/l), uric acid at 34. 7mg/l, phosphorus level 153mg/l, corrected calcium level 74 mg/l, creatinine 18mg/l (Normal value 6–12mg/l) and urea 0.54g/l (Normal value 0.35–0.55g/l). The haemostasis workup found a prothrombin rate of 54% with an extended partial thromboplastin time of 35s with a 24s tempo.

An infectious workup was completed, including an ECBU, and a blood culture that came back negative. A *trans*-thoracic echocardiography was performed, showing a circumferential pericardial effusion of moderate size with an LVEF of 57%.

In front of this picture of shock associated with a generalized edema with hypoalbuminia with a peritoneal effusion without notion of renal loss of protein, or hepatic or cardiac insufficiency, as well as the presence of a tumoral etiology, the diagnosis of a secondary cappilar hyperpermeability syndrome is made.

The patient was put under oxygen therapy under high concentration mask with a flow rate of 15l/min, filling with 0.9% isotonic saline with the introduction of vasoactive inotropes, as well as the introduction of a cytoreductive treatment.

The evolution was marked by the worsening of the hemodynamic and respiratory instability, along with the installation of a consciousness disorder requiring the use of an invasive ventilation, but unfortunately, the patient presented a refractory cardio-respiratory arrest that was not successfully recovered 24 hours after his admission.

## 3 Discussion

Clarkson's syndrome or capillary hyperpermeability syndrome was first reported in 1950 by Bayard Clackson and colleagues, hence its name. Since then, about 500 cases have been reported in the literature, mainly in young adults, but also 32 cases have been reported in children, such as ours [1,2].

Clarkson syndrome in its pediatric form affects newborns, infants, pre-school children and mainly school children, and is often preceded by a viral disease [1].

It is a disease whose pathophysiology remains unclear, characterized by a sudden increase in the permeability of the vascular sector responsible for a leakage of proteins to the interstitial space causing edema.

Clinically, three phases have been described in the literature, especially the prodromal phase, which is oligosymptomatic and characterized by nonspecific signs such as flu-like syndrome, lasting from 1 to 4 days, followed by the capillary leak phase, which is marked by the hypovolemic shock associated with a state of anasarca due to the extravasation of more than 70% of the plasma fluid into the interstitial space, this phase can last from 1 to 5 days [3,4]. Finally, the post-leak phase which is marked by the remobilization of plasma fluid into the intravascular environment: a decrease in edema, normalization of arterial pressure, and diuresis, to be noted that during this phase the patient is at high risk of a cardiovascular overload [3,4].

The diagnosis is made after the elimination of the disease, which should be evoked in front of a recurrent appearance of a generalized edematous syndrome associated with a triad of hypo albuminemia, hemoconcentration and hypo perfusion [1], an electrophoresis of plasma proteins most often reveals a monoclonal gammapathy type IgG in 68%–85% of adults, while it is often absent in children [[5], [6], [7]].

The capillary leakage syndrome is usually secondary to an underlying pathological condition or a drug intake as it can be idiopathic, clarkson disease. Drug-induced cases are frequently found in patients undergoing anti-tumor treatment, mainly interlukine 2, interferon, rituximab, Anti-PD1 [7], retinoic acid or differentiation syndrome in induction therapy in acute promyelocytic leukemia, Ovarian hyperstimulation syndrome, phagocytic lymphohistiocytosis [7,8], it is also found in hematological malignancies essentially and more rarely in solid tumors, we can also find as secondary causes, autoimmune diseases and viral infections. Idiopathic clarkson's syndrome is often under-diagnosed in front of sepsis, which makes the estimation of its incidence difficult [7].

Given the rarity of the syndrome, the treatment is based on essentially theoretical considerations. Volume expansion based on crystalloids remains the first-line treatment, with close monitoring of hemodynamic constants, albumin can also be administered if filling is ineffective, as well as steroids, especially during the capillary leak phase, which have been reported to be effective in the majority of causes of capillary leak syndrome [7,9]. In order to avoid recurrence, the administration of intravenous high doses of Ig should be considered during the first 4 days, 2g/kg/day and then 2g/kg/month until continuous remission is obtained [5,7]. Theophylline and terbutaline are also used for preventive purposes by increasing the production of cAMP and by diminishing the capillary permeability [6], but it has recently been reported that their long-term efficacy remains limited in 84% of patients, as a therapeutic means. We note that anti TNF alpha are used in idiopathic recurrent forms in children [7].

In our case, Clarkson's Syndrome secondary to acute leukemia was evoked in front of the recurrence of the edematous syndrome, clinically the state of shock associated with the anasarca, the high abundance of the pericardial effusion, biologically the hypo albuminemia, the proteinuria which came back negative, and the absence of an affection explaining the edemas.

Unfortunately, the patient has quickly worsened his respiratory distress along with a hypovolemic shock which was resistant to the filling, then cardio respiratory arrest, the child did not recover even with resuscitation measures.

The SCARE guidelines were used in the writing of this paper [10].

## 4 Conclusion

Given the rarity and fatality of Clarckson Syndrome, few cases are reported in the literature, which makes management difficult since it is based only on theoretical data, hence the interest in researching it in the case of shock associated with oedemas.

## Consent for publication

Written informed Consent was obtained from the Child's patients for publication of this case report and accompanying images. A copy of the written consent is available for review by the Editor-in-Chief of this journal on request.

## Provenance and peer review

Not commissioned, externally peer reviewed.

## Ethical approval

The ethical committee approval was not required give the article type (case report).However, the written consent to publish the clinical data of the patients was given and is available to check by the handling editor if needed.

## Sources of funding

None.

## Author contribution

HAJAR BENZEKRI: study concept or design, data collection, data analysis or interpretation, writing the paper, AMINE BOUCHLARHEM: Data collection, data analysis, NOUR EL HOUDA LAMASSAB: Data collection, data analysis, MERYEM JABRI: Data collection, data analysis, FADOUA JEBROUNI: Data collection, data analysis, CHAIMAE DAOUDI: Data collection, data analysis, NOUREDINE OULALI: supervision and data validation.

## Registration of research studies

This is not an original research project involving human participants in an interventional or an observational study but a case report. This registration is was not required.

## Guarantor

HAJAR BENZEKRI.

## Declaration of competing interest

None.
